# Improving communication of clinical trial results: plain English summaries in the NIHR journals library

**DOI:** 10.1186/1745-6215-16-S2-P192

**Published:** 2015-11-16

**Authors:** Emma Kirkpatrick, Liz Brindley, Elaine Williams

**Affiliations:** 1 National Institute for Health Research Evaluation, Trials and Studies Coordinating Centre, Southampton, UK; 2 University of Southampton, Southampton, UK

## Background

Plain English summaries (PESs) are a valuable tool for making research findings available and accessible to patients and the public. They provide brief descriptions of research written in non-specialist language. The National Institute for Health Research Journals Library (NIHRJL) currently requires authors to provide a 250 word PES. This is published online as part of the final, open-access report. Practice varies among other funders and journals, some of whom employ independent external writers (IEWs) for this purpose.

## Objectives

To compare ease of reading and understanding of PESs written by clinical trial teams and an IEW.

## Methods

Forty reports undergoing the editorial process for the NIHRJL will be included in the study. The PES for each report will be revised by authors in line with detailed guidance and edited by an IEW.

## Results

Ease of reading will be measured using the Flesch Reading Ease score. Scores range from 0-100; higher scores indicate text that is easier to read. Ease of understanding will be assessed by asking public reviewers to read a selection of PESs and rate them from 1 (did not understand) to 4 (understood all). Statistical comparison of group scores will be reported.

## Discussion

High quality PESs are a priority for dissemination of clinical trial results to patients and the public but it is currently unclear who is best placed to produce them. Results of this study will inform future practice for the NIHRJL and will be relevant to all agencies communicating trial findings to patients and the public.

